# Who's driving? The default mode network in healthy elderly individuals at risk of cognitive decline

**DOI:** 10.3389/fneur.2022.1009574

**Published:** 2022-11-30

**Authors:** Mauricio González-López, Eduardo Gonzalez-Moreira, Ariosky Areces-González, Deirel Paz-Linares, Thalía Fernández

**Affiliations:** ^1^Departamento de Neurobiología Conductual y Cognitiva, Instituto de Neurobiología, Universidad Nacional Autónoma de México, Querétaro, Mexico; ^2^Center for Biomedical Imaging and Neuromodulation, Nathan Kline Institute for Psychiatric Research, Orangeburg, NY, United States; ^3^MOE Key Lab for Neuroinformation, The Clinical Hospital of Chengdu Brain Science Institute, University of Electronic Science and Technology of China, Chengdu, China; ^4^Faculty of Technical Sciences, University of Pinar del Río “Hermanos Saiz Montes de Oca, ” Pinar del Rio, Cuba; ^5^Neuroinformatics Department, Cuban Neuroscience Center, Havana, Cuba

**Keywords:** healthy aging, EEG connectivity, default mode network, cognitive decline, BC-VARETA, functional connectivity

## Abstract

**Introduction:**

Age is the main risk factor for the development of neurocognitive disorders, with Alzheimer's disease being the most common. Its physiopathological features may develop decades before the onset of clinical symptoms. Quantitative electroencephalography (qEEG) is a promising and cost-effective tool for the prediction of cognitive decline in healthy older individuals that exhibit an excess of theta activity. The aim of the present study was to evaluate the feasibility of brain connectivity variable resolution electromagnetic tomography (BC-VARETA), a novel source localization algorithm, as a potential tool to assess brain connectivity with 19-channel recordings, which are common in clinical practice.

**Methods:**

We explored differences in terms of functional connectivity among the nodes of the default mode network between two groups of healthy older participants, one of which exhibited an EEG marker of risk for cognitive decline.

**Results:**

The risk group exhibited increased levels of delta, theta, and beta functional connectivity among nodes of the default mode network, as well as reversed directionality patterns of connectivity among nodes in every frequency band when compared to the control group.

**Discussion:**

We propose that an ongoing pathological process may be underway in healthy elderly individuals with excess theta activity in their EEGs, which is further evidenced by changes in their connectivity patterns. BC-VARETA implemented on 19-channels EEG recordings appears to be a promising tool to detect dysfunctions at the connectivity level in clinical settings.

## Introduction

As life expectancy has increased in recent years, so has the prevalence of neurocognitive disorders—formerly known as mild cognitive impairment and dementia—since age is the main risk factor for the development of these disorders. Neurocognitive disorders are classified according to their etiology, with Alzheimer's disease (AD) being the most common (1). The physiopathological features of AD may develop in individuals from years to decades before the onset of clinical features (2), and beta-amyloid (βA) depositions can be found in 20–50% of healthy and cognitively normal older adults (3, 4). Given that early interventions that take place before clinical signs appear are more effective, research on the early detection of AD or the identification of biomarkers with good predictive power has become increasingly important.

The National Institute on Aging and Alzheimer's Association (NIA-AA) has released recommendations for the detection of biomarkers during the preclinical stage of the disease, which include Pittsburgh Compound B positron emission tomography (PIB-PET), cerebrospinal fluid (CSF) phosphorylated tau concentrations and evaluating the degree of gray matter atrophy using magnetic resonance imaging (MRI) (5, 6). However, even though the use of these tools has been validated and they are recommended for research, they have limited value in everyday clinical settings, especially in developing countries, due to their elevated costs or their invasive nature.

A non-invasive and cost-effective tool to assess brain function is the electroencephalogram (EEG), which measures the electrical activity of the brain with surface electrodes placed on the subject's scalp. EEG provides information about brain function with high temporal resolution and has proven to be a useful tool in the study of neuropsychopathology, since the recorded electrical activity reflects the overall interaction between multiple groups of neurons. As research on the preclinical phase of AD and other dementias has received increasing attention, so has the validation of EEG biomarkers (7).

A quantitative analysis of the EEG (qEEG) may be performed to determine the power, among other measures, of specific discrete frequencies (or frequency bands) and can be used to compare these values to a normative database to establish whether the EEG of a certain subject deviates from their age-expected values. The qEEG, therefore, appears to be a promising tool for predicting future cognitive decline in healthy elderly individuals (8–10), and future progression to dementia in patients with MCI (11–13). Moreover, using variable resolution electromagnetic tomography (VARETA), Prichep (14) reported that in healthy older adults that declined over the course of 7–10 years, the most likely current sources of the most abnormal narrow band within the theta frequency range were the hippocampus, parahippocampal gyrus, amygdala, and parietotemporal cortex; however, this study did not present the results of other frequency bands. Musaeus et al. (10) have also suggested that theta activity is positively related to cognitive decline in a sample of almost 400 subjects. Further research has established that low-frequency activity in the EEG is related to AD-CSF biomarkers in both healthy participants (15) and participants with dementia (16).

Considering this evidence, our research group has hypothesized that an excess of theta activity in the EEG might distinguish between two subpopulations of healthy elderly individuals, one of which may be developing a pathological process that is currently subclinical in nature. We have reported that these individuals with an excess of theta activity present atypical patterns in event-related potentials compared to those of a control group with normal EEGs in tasks involving semantic processing (17) and inhibitory control (18), as well as changes in cortical volume using MRI (19), which we have suggested to be a compensatory mechanism.

Since the brain is organized into functional networks and AD has long been considered a “disconnection syndrome” (20, 21), the study of EEG activity should go beyond the characterization of independent regions. Numerous functional connectivity studies using magneto/electroencephalography (M/EEG), have found differential changes along the AD continuum [(22–24); See Lejko et al. (25) for a recent review and meta-analysis regarding alpha connectivity]. Of particular interest for the present study is the default mode network (DMN), which involves several brain areas that are inhibited during the performance of tasks that involve attention to external stimuli (26) and has been linked to episodic memory and internally focused tasks [for a recent review of the DMN, see (27)]. Research has shown that the functional connectivity (FC) of the DMN changes as a function of age (28) and that this FC is further diminished in MCI and AD (29–32). Moreover, the anatomical regions of the DMN overlap with the typical distribution of depositions of βA, a physiopathological feature of AD, and this βA burden is related to the FC of the DMN (3, 33, 34) and here have been reports of reduced DMN deactivation during tasks in APOE e4 carriers (35, 36).

Most studies on FC are conducted using the analysis of blood oxygenation level-dependent (BOLD) signals detected by functional magnetic resonance imaging (fMRI). This approach can reflect the neural correlates (responses and connectivity) of brain function during the execution of tasks or during the resting state. Additionally, diffusion tension imaging (DTI) based on MRI allows the extraction of probabilistic maps of long-range connectivity due to white matter tracts. These are the gold-standard methods that can, non-invasively, investigate brain connectivity with sufficient spatial resolution to reach the columnar level, providing reliable correlates of spatially distributed neural activity. Without undermining the usefulness and advantages of these techniques, it is important to emphasize that they do not directly reflect neural dynamics or synaptic transmission. The BOLD signal is a consequence of a slow (seconds-long) metabolic/hemodynamic cascade, which is correlated with synaptic activity. Thus, it does not reach the millisecond temporal resolution of faster brain rhythms. DTI, on the other hand, provides structural probabilistic maps of plausible connections based on the diffusion of water across white matter tracts but cannot precisely reveal the pathways that influence neural communication.

Noninvasive M/EEG recordings may account for these limitations and bridge the gap between other slower and indirect imaging methods, i.e., the previously mentioned fMRI. Its direct link to local field potentials (associated with synaptic events) and high temporal resolution (on the order of milliseconds) allows the tracking of neural processes underlying human perception and cognition (37–39). The local field potential of synchronized neural activity within neural regions (generators) creates a non-invasive, observable primary current density (PCD). An accurate estimation of the PCD given these signals thus provides a representation of the neural dynamics. Therefore, a M/EEG-based connectivity analysis constitutes a strong approach to study brain functional networks in the resting state (40, 41).

This paper targets the frequency-specific analysis of the EEG source activity and functional connectivity with the novel BC-VARETA toolbox (42). The frequency-specific analysis is based upon cross-spectra, which summarize all the second-order multivariate statistical properties of source activity at each frequency, including the functional connectivity. BC-VARETA achieves increased spatial resolution due to direct Bayesian inference with cross-spectral, a priori probabilities targeting these source cross-spectra from the EEG signals (42, 43).

The present study aims to explore the feasibility of functional connectivity under aggravating conditions for the spatial resolution of EEG source analysis commonly found in clinical settings, i.e., the recording of the EEG with only 19 electrodes. Consequently, this feasibility is explored by the detection of differences in terms of functional connectivity as defined by the Phase Slope Index (PSI) of the DMN between a group of healthy participants at risk of cognitive decline (i.e., with excess theta absolute power) and a control group of healthy elderly individuals with normal EEG.

## Materials and methods

This study was approved by the Bioethics Committee of the Institute of Neurobiology of the National Autonomous University of Mexico (INEU/SA/CB/109) in accordance with the Declaration of Helsinki.

### Participants

This study was conducted with a total of 215 healthy volunteers aged 60 or older, which were recruited from the general population by different means such a radio announcements, flyers, and word-of-mouth recommendations from other participants. The participants were interviewed and assessed to verify whether they fulfilled the inclusion criteria. The inclusion criteria were: (1) right-handedness, (2) no history of neurological conditions, (3) no presence of psychiatric disorders, (4) blood analysis within healthy ranges for glucose, cholesterol, triglycerides, hemoglobin, and thyroid-stimulating hormone, (5) a minimum of 9 years of formal education and (6) an IQ above 80, which was assessed using the Wechsler Adult Intelligence Scale, Fourth Edition (WAIS-IV). Moreover, a gerontopsychiatrist evaluated the participants, and only individuals with a score of 1 or 2 on the Global Deterioration Scale (44), indicating no objective evidence of cognitive decline, were included. A sample size calculation was conducted using G^*^ Power (45) considering previous research from our group. The means and standard deviations for the highest z-score of absolute theta power were obtained for each of two groups, which were defined in the same way as in the present study (see below). For an estimated effect size of d = 2.904 (Mean_1_ = 0.684, S.D._1_ = 0.543; Mean_2_ = 3.168, S.D._2_ = 1.081), a significance level set to α = 0.05 and a statistical power of 1-β = 0.95, the sample size analysis yielded a result of 5 subjects per group.

After the fulfillment of the inclusion criteria was verified, an EEG was recorded (see below). The participants with an excess of theta activity (z > 1.96) in at least one lead were assigned to the risk group (RG; *n* = 30). The individuals that exhibited a normal EEG both in qualitative and quantitative terms, that is, no abnormal waveforms were observed, and the z scores of all their quantitative broad-band measures were within −1.64 and 1.64, were assigned to the control group (CG; *n* = 30). The participants that exhibited an abnormality different from an excess of theta activity were not included in this study. Out of the total sample, 60 participants met the inclusion criteria for this study (*n* = 60, mean age, 67.58; standard deviation, 4.61; 38 women).

All participants signed a written informed consent form. The groups did not differ in terms of sex, age, years of education or IQ (see Table 1).

**Table 1:** Demographic and psychometric assessments of intelligence of the control group (CG) and the risk group (RG) according to the WAIS-IV.

	**CG**	**RG**	**Tests statistic**	***p***
*N*	30	30		
Sex	21 f / 9 m	17 f/13 m	χ^2^ (1) = 1.15	0.28
Family history of dementia	26 n / 4 y	26 n / 4 y	χ^2^ (1) = 0.00	1.00
GDS score	26(1)/4(2)	25(1)/5(2)	χ^2^ (1) = 0.13	0.72
Age	66.9 (4.4)	68.26 (4.78)	*t* (58) = 1.15	0.25
Years of education	15.73 (4.09)	14.7 (2.92)	*t* (58) = 1.13	0.26
VCI	122.8 (9.02)	124.26 (7.89)	*t* (58) = 0.66	0.51
PRI	105.73 (13.42)	105.40 (13.52)	*t* (58) = 0.09	0.92
WMI	105.33 (8.57)	106.56 (6.99)	*t* (58) = 1.11	0.27
PSI	109.5 (17.68)	105.1 (16.12)	*t* (58) = 1.01	0.31
FSIQ	104.16 (21.13)	107.6 (10.53)	*t* (58) = 0.79	0.43

Data are presented as means and standard deviations. VCI, verbal comprehension index; PRI, perceptual reasoning index; WMI, working memory index; PSI, processing speed index; FSIQ, full scale IQ.

### Electroencephalography (EEG)

Digital EEGs were recorded in participants at rest with their eyes closed using a MEDICID IV system (Neuronic Mexicana, SA, Mexico) and TrackWalker^TM^ version 2.0 software, with 19 silver electrodes in the International 10–20 system mounted on an elastic cap (Electro-Cap, International Inc., Eaton, Ohio, USA) and referenced to linked earlobes. The participants were seated in a comfortable chair inside a faradized, sound-proofed, dimly lit room. The EEG was digitized at a sampling rate of 200 Hz, and the signal was amplified with a gain of 20,000. The impedance of each electrode was kept below 10 kOhm. The EEG was recorded for 10 min during the eyes-closed condition. None of the participants exhibited paroxysmal abnormalities. None of the participants were under the effects of any psychopharmacological drug and were instructed not to take any substance that could induce drowsiness the night before the recording. During the recording, they were instructed to keep their eyes closed and remain relaxed. The subjects were also cautioned not to fall asleep.

**Figure 1:**
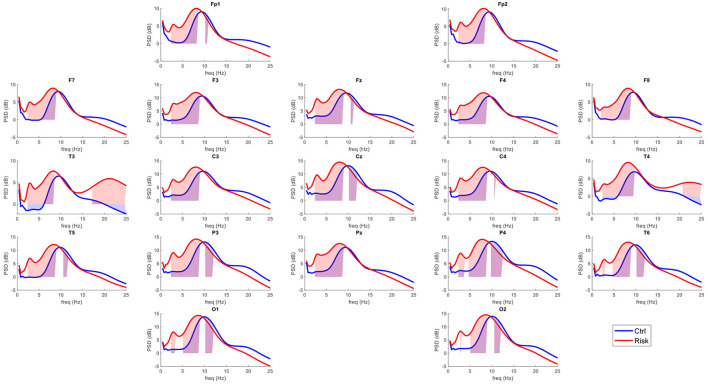
Average power spectra per group in the surface level EEG. In red, average power spectra across the 19 leads of the International 10–20 system for the risk group (RG). In blue, average power spectra for the control group (CG). The shaded areas represent the frequencies that exceeded the probability threshold (*p* = 0.05).

Twenty-four artifact-free epochs of 2.56 s each were selected from each recording by an expert in electroencephalography. The expert selected EEG segments without intrinsic or extrinsic artifacts in any channel. The selected segments throughout the recording had to maintain the frequency and amplitude characteristics of the posterior dominant rhythm to avoid any activity associated with drowsiness. The qEEG analysis was performed offline using a fast Fourier transform, and cross-spectral matrices were calculated with a frequency resolution of 0.3906 Hz. Absolute and relative power values were obtained for each frequency band (delta [0.5–3.5 Hz], theta (3.5–7.5 Hz], alpha1 (7.5–10 Hz], alpha2 (10–12.5 Hz], beta1 (12.5–15.5 Hz], beta2 (15.5–20 Hz], and beta3 (20–25 Hz]). The geometric power was subtracted for each individual cross-spectrum. This correction consisted of a rescaling of the power spectrum that helps to reduce variance not related to physiological factors up to 42% (46), and z scores for each measure of absolute power (z-AP) were obtained by comparing the raw data to a normative database (47). The average power spectra for each group at each of the 19 leads are shown in Figure 1.

### Analysis of the source activity and functional connectivity from the EEG data

Consider the EEG source activity **ι** (*t*), the vector time series whose entries **ι**( *s,t*) comprise the currents at each source location *s* and time *t*. Consider then EEG data **v** (*t*), the vector time series whose entries **v** (*e,t*) comprise the electric potential at each electrode location *e* and time *t*. We estimate functional connectivity of this source activity **ι** (*t*) by definition of multivariate statistics of the vector time series (48). This estimation involves the inverse solutions from EEG data **v** (*t*), by definition of solutions to an ill-posed inverse problem in obtaining EEG source activity **ι** (*t*) (49).

Formalizing an inverse problem is linear forward model (Equation 1), as expressed by the lead field matrix **L** whose entries **L** (*e, s*) factor the activity **ι** (*s,t*) at each source location *s* to explain their data **v** (*e, t*) at each electrode location *e* (39, 50, 51). Complementing this forward model are residuals **ξ** (*t*), or vector time series which add onto the data **v** (*t*) which is not explained by lead field **L** form the source activity **ι** (*t* ).


(1) v(t)= Lι(t)+ξ(t);∀t

From the literature for this inverse problem (Equation 1) (52) formalizing an inverse solution could be the linear inverse model (Equation 2), as expressed by the pseudo-inverse **T** (to the matrix **L**) whose entries **T** (*s, e*) factor the data **v** (*e, t*) at each electrode location *e* to explain the activity **ι** (*s, t*) at each source location *s*. From the general literature of inverse problems, pseudo-inverse **T** is the proximal operator or back projector that solves an optimization problem (53–56).


(2) ι(t)= Tv(t);∀t

This pseudo-inverse **T** (Equation 2) optimizes the cost function *R*(**ξ**(*t*);∀*t*) which is upon the reminders of source activity **ι**(*t*) explaining the data **v**(*t*). Resolving the inverse problem with an ill-posed nature which is due to the ill-condition of matrix **L** (Equation 1) (57) is the Tikhonov regularization or constrained optimization which imposes over the inverse solutions penalization function *P*(**ι**(*t*);∀*t*) (58). The target function of constrained optimization is *Q*(**T**, **v**(*t*);∀*t*) (Equation 3) which in addition redefines functions *R*(**ξ**(*t*);∀*t*) and *P*(**ι**(*t*);∀*t*) according to the previous forward model (Equation 1) and inverse model (Equation 2).


(3) $$\;T\left(v\left(t\right);\forall t\right)=argmin_{T}\left\{Q\left(T,v\left(t\right);\forall t\right)\right\}$$

(4) Q(T,v(t);∀t)=R((I−LT)v(t);∀t)+P(Tv(t);∀t)

We opt for a formalization of the inverse problem (Equation 1) and inverse solution (Equation 2) in the frequency domain of the time series which aims to obtain the specific patterns of rhythmic source activity (59–63). A target function (Equations 3, 4) is defined upon cross-spectrum, an essential statistic in the frequency domain of stationary time series (64). Table 2 summarizes the basic definitions to reproduce our analysis, including quantities (Table 2, 1–3), models (Table 2, 4–8), method (Table 2, 9–11), and functional connectivity (Table 2, 12, 13).

**Table 2:** Summary of the qEEG analysis.

**Quantity**	**Source**	**Data**
1. Time series	ι_*m*_(*t*);*m* = 1…*M*	**v**_*m*_(*t*);*m* = 1…*M*
2. Fourier transform	ι_*m*_(*f*);*m* = 1…*M*	*v*_*m*_(*f*);*m* = 1…*M*
3. Cross-spectrum	$\Sigma_{u}\left(f\right)=\left(\Sigma_{\left(m=1\right)}^{M}\iota_{m}\left(f\right)\iota_{m}^{†}\left(f\right)\right)/M$	$\Sigma_{vv}\left(f\right)=\left(\Sigma_{\left(m=1\right)}^{M}v_{m}\left(f\right)v_{m}^{†}\left(f\right)\right)/M$
**Model**		
4. Forward	*v*_*m*_(*f*) = *Lι*_*m*_(*f*)+ξ_*m*_(*f*)	
5. Inverse	ι_*m*_(*f*) = *Tv*_*m*_(*f*) also $\Sigma_{u}\left(f\right)=T\Sigma_{vv}\left(f\right)T^{†}$	
6. Reminders	ξ_*m*_(*f*) = (*I*−*LT*)*v*_*m*_(*f*) also $\Sigma_{\xi \xi }\left(f\right)=\left(I-LT\right)\Sigma_{vv}\left(f\right){\left(I-LT\right)}^{†}$	
7. Cost function	*R*(**Σ**_**ξ*ξ***_(*f*)) = *tr*(**Σ**_**ξ*ξ***_(*f*))	
8. Penalization function	$P\left(\Sigma_{\iota \iota }\left(f\right)\right)=\alpha_{1}\left(f\right)tr^{\frac{1}{2}}\left(\Sigma_{\iota \iota }\left(f\right)\right)+\alpha_{2}\left(f\right)tr\left(\Sigma_{\iota \iota }\left(f\right)\right)$	
**Method**		
9. Target function	$Q\left(\text{T},\Sigma_{vv}\left(f\right)\right)=R\left(\left(\text{I}-\text{L}\text{T}\right)\Sigma_{vv}\left(f\right){\left(\text{I}-\text{L}\text{T}\right)}^{†}\right)+P\left(\text{T}\Sigma_{vv}\left(f\right){\text{T}}^{†}\right)$	
10. Inverse solution	**T**(**Σ**_*vv*_(*f*)) = *argmin*_**T**_{*Q*(**T**, **Σ**_*vv*_(*f*))}	
11. Cross-spectrum	$\Sigma_{\iota \iota }\left(f\right)=\text{T}\left(\Sigma_{vv}\left(f\right)\right)\Sigma_{vv}\left(f\right){\text{T}}^{†}\left(\Sigma_{vv}\left(f\right)\right)$	
**Functional connectivity**		
12. Coherence	${\text{C}}_{\iota \iota }\left(s,s^{{\;}^{\prime }},f\right)=\frac{\Sigma_{\iota \iota }\left(s,s^{{\;}^{\prime }},f\right)}{\sqrt{\Sigma_{\iota \iota }\left(s,s,f\right)\Sigma_{\iota \iota }\left(s^{{\;}^{\prime }},s^{{\;}^{\prime }},f\right)}}$	
13. Phase Slope Index	${\text{K}}_{\iota \iota }\left(s,s^{{\;}^{\prime }},f_{0}\right)=Im\left(\underset{f\varepsilon_{F}}{\sum }{\text{C}}_{\iota \iota }\left(s,s^{{\;}^{\prime }},f\right)⊙{\text{C}}_{\iota \iota }^{*}\left(s,s^{{\;}^{\prime }},f+df\right)\right)$	

For the type of rhythmic source activity, multivariate statistics in the frequency domain of vector time series **ι** (*t*) define the functional connectivity (65). Our definition bases upon the Fourier transform **ι** (*f*) whose entries **ι** (*s, f*) represent the amplitude and phase of these rhythms at each source location *s* and frequency *f*. The sample space for these statistics are independent realizations of the source activity **ι**_*m*_ (*t*)'s (Table 2, 1) that produce Fourier transform **ι**_*m*_ (*f*)'s (Table 2, 2).

The source cross-spectrum **Σ**_*ιι*_ (*f*) (Table 2, 3), estimates the second-order statistic the Fourier transform **ι**_*m*_ (*f*)'s by the definition of Hermitian covariance matrix (66–68). This cross-spectrum **Σ**_**ιι**_ (*f*) is sufficient statistic for the type of stationary time series emerging during resting state or task in a block design from the DMNs (68–70).

Estimation of **Σ**_*ιι*_ (*f*) is more precise with direct (cross-spectral) inverse solution from the EEG cross-spectrum **Σ**_**vv**_ (*f*), also sufficient statistic for independent realizations of the data **v**_*m*_(*t*)'s that produce Fourier transform **v**_*m*_ (*f*)'s (Table 2, 1–3). Achieving this cross-spectral inverse solution are the forward model (Table 2, 4) and the inverse model (Table 2, 5) in frequency domain. The lead field matrix **L** and pseudo-inverse **L** in these models project forward and backward between the source **ι**_*m*_ (*f*)'s and their data **v**_*m*_ (*f*)'s frequency specific patterns of amplitude and phase.

A cost function *R*(**Σ**_**ξξ**_ (*f*)) is upon the residuals **ξ**_*m*_ (*f*) (Table 2, 6) as defined by the trace operator of their cross-spectrum **Σ**_**ξξ**_ (*f*) (Table 2, 7). Complementing this cost function is a penalization function *P*(**Σ**_*ιι*_ (*f*)) (Table 1, 8) as defined by the Elastic Net nuclear quasinorm, linear combination of square root trace and trace operators of the cross-spectrum **Σ**_*ιι*_ (*f*). Elastic Net nuclear quasinorm pursues structured sparsity of the cross-spectrum topographic projection $\left(\text{spectrum }{\hat{\sigma }}_{\iota \iota }^{2}\left(f\right)=diag\left({\hat{\Sigma }}_{\iota \iota }\left(f\right)\right)\right)$ ameliorating extreme distortions due to ill-condition of the EEG Lead field matrix **L**.

Constrained optimization is for target function *Q*(**T**, **Σ**_**vv**_ (*f*)) (Table 2, 9) minimized by pseudo-inverse **T**(**Σ**_**vv**_ (*f*)) (Table 2, 10) defined as matrix function of the data cross-spectrum **Σ**_**vv**_ (*f*) which provides cross-spectral inverse solution **Σ**_*ιι*_ (*f*) (Table 2, 11). From this cross-spectrum **Σ**_*ιι*_ (*f*) at each frequency *f* we first determine the coherence **C**_*ιι*_ (*f*) (Table 2, 12) and second the functional connectivity **K**_*ιι*_ (*f*_0_) (Table 2, 13) as defined by the Phase Slope Index (PSI) of each frequency band (delta, theta, alpha, beta). For these frequency bands *f*_0_ represents the starting frequency and Δ*f*_0_ the band width employed to determine the PSI or functional connectivity **K**_** *ιι***_ (*f*_0_ ).

Defining functional connectivity for the DMN is here from the matrix entries $K_{\iota \iota }\;\left(s,s^{\prime },\;f_{0}\right)$ averaged over the rows *s* and columns *s*′ that correspond to cortical regions. These regions are described by Buckner et al. (26) and have been used in numerous studies involving the DMN. The selected anatomical regions were the dorsomedial prefrontal cortex (dMPFC), posterior cingulate and retrosplenial cortex (PCC/Rsp), inferior parietal cortex (IPC), lateral temporal cortex (LTC), ventromedial prefrontal cortex (vMPFC) and hippocampal formation (HF+), which includes the entorhinal and parahippocampal cortices.

### Statistical analysis

The statistical analyses used in this study were based on a non-parametric method, permutation-based statistics tests (71). The major advantages of the permutation-based framework for electrophysiology data analysis are related to the lack of assumptions as to parametric data distribution and the ability to appropriately conduct multiple comparisons controlling for Type I error (72, 73). Therefore, for permutation-based tests, the statistical distribution is not based on a formula (wherein statistical significance is based on assumptions as to the distribution). Instead, the statistical distribution is obtained empirically from the data by simulating situations that could have arisen under the null hypothesis with the data obtained (significance based on data). To build up each empirical null hypothesis distribution, we used 5,000 random permutations. The statistical significance was assessed by counting the number of null hypothesis tests (permutations) values higher than the observed statistic test value and dividing it by the total number of null hypothesis tests (number of permutations). The permutation-based test does not require a specific null hypothesis distribution such as the Gaussian or F-distribution. The significance level to reject the null hypothesis was set to *p* = 0.05. Finally, a multiple comparison correction was applied. One commonly used approach for this end is the Bonferroni correction which is based on dividing the *p*-value by the number of tests carried out. However, even when Bonferroni correction is suitable in some brain data analyses like hypothesis-driven tests, it is not appropriate for time-frequency decomposition results where many tests are performed over time points, frequency bins, and ROIs (74). The Bonferroni correction assumes that the tests are independent, which is not the case for brain data, where a strong correlation is found among the time series points, frequency bands, and neighboring neural populations. In this work, we applied the pixel-based multiple comparisons correction. Correcting for multiple comparisons using pixel-based statistics relies on creating a distribution of the pixel from each iteration of permutation testing with the most extreme statistical value.

## Results

**Figure 2:**
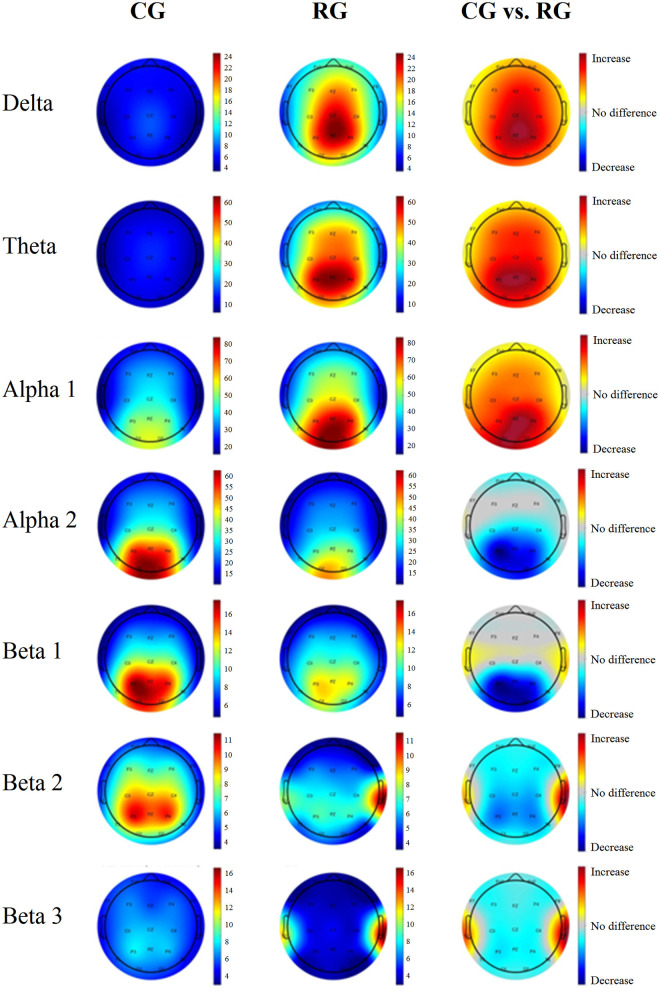
Average power maps per group and probability maps of the differences in the surface level EEG. The first column corresponds to the CG, and the middle column corresponds to the RG. The last column corresponds to the significance values from the comparison between the groups. Warm colors indicate that the risk group AP values are higher than those of the control group. Cool colors represent lower AP values in the risk group than in the control group. Only the areas that exceeded the probability threshold (*p* = 0.05) are colored. The rows represent each frequency band.

Figure 1 shows the power spectra across the 19 leads for each group. This indicates a general slowing of the EEG signal in healthy participants considered at risk for cognitive decline. The qEEG maps shown in Figure 2 illustrate the probability values associated with each comparison at each frequency band.

**Figure 3:**
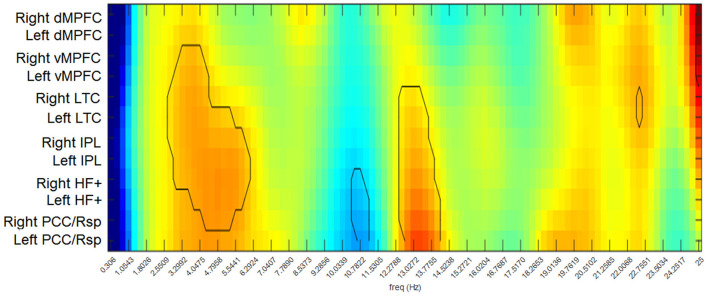
Differences between groups in functional connectivity strengths for each node of the DMN. Warm colors indicate higher values in the RG than in the CG. Cool colors indicate higher values in the CG than in the RG. The contour indicates the comparisons that exceeded the probability threshold (*p* = 0.05).

The differences between the RG and the CG in functional connectivity strength across the analyzed frequencies are shown for each node of the DMN in Figure 3. This connectivity strength represents the relation of each node with all the other nodes in the network. The participants in the RG exhibited significantly higher values of connectivity than those in the CG for most of the DMN nodes in the delta and theta band frequencies, except for the bilateral dMPFC and the left PCC/Rsp. Similarly, the RG displayed higher connectivity strength in the beta1 band for all the nodes, except for the medial prefrontal cortices. In contrast, the RG showed significantly lower values of connectivity strength in the bilateral HF+ and PCC/Rsp in the alpha2 band.

**Figure 4:**
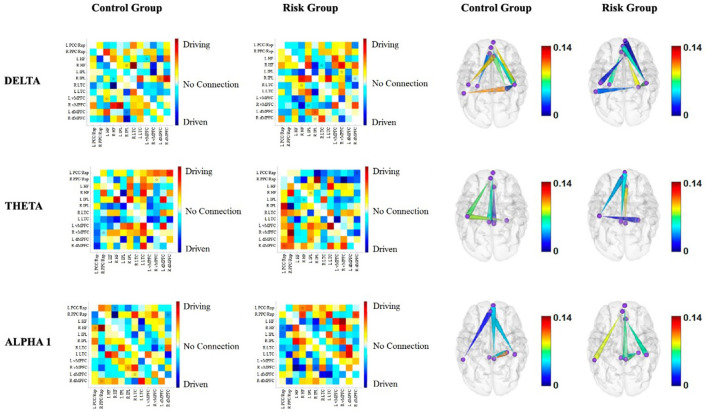
Functional connectivity between the nodes of the DMN for each group for delta, theta and alpha1. The first two columns correspond to the functional connectivity matrices of the CG and the RG, respectively. Warm colors indicate the driving nodes, and cool colors indicate the driven nodes. The connections that exceeded the probability threshold (*p* = 0.05) are indicated with a star and are shown in the cortical maps of the last two columns. Direction is represented by the cones, with the vertices indicating the driven nodes and the bases indicating the driver nodes. The color scale illustrates the connectivity strength. The rows represent each frequency band.

**Figure 5:**
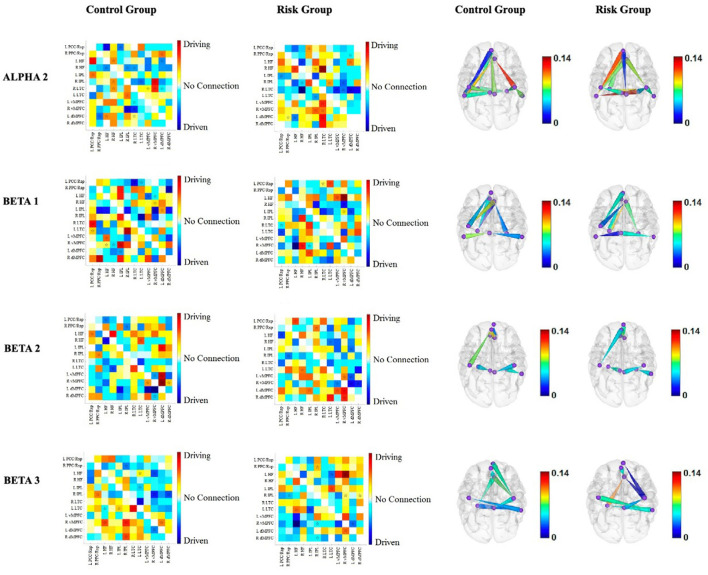
Functional connectivity between the nodes of the DMN for each group for alpha2, beta1, beta2, and beta3. The first two columns correspond to the functional connectivity matrices of the CG and the RG, respectively. Warm colors indicate the driving nodes, and cool colors indicate the driven nodes. The connections that exceeded the probability threshold (*p* = 0.05) are indicated with a star and are shown in the cortical maps of the last two columns. Direction is represented by the cones, with the vertices indicating the driven nodes and the bases indicating the driver nodes. The color scale illustrates the connectivity strength. The rows represent each frequency band.

The results of the directionality of functional connectivity for each group, as well as cortical maps representing the direction of the connections that differed between the groups, are shown in Figures 4, 5. Most of the differences consisted in opposite directions of the information flow between pairs of nodes of the DMN. The results are briefly described for each frequency band below:

**Delta band**. Intrahemispheric connectivity differences appear to be more pronounced within the right hemisphere. Moreover, the left hemisphere appears to be a driver of right nodes in the RG, whereas the opposite is true for the CG (Figure 4).

**Theta band**. Interestingly, prefrontal nodes of the DMN appear to be drivers of posterior nodes in the RG, with higher connectivity strengths within the right hemisphere (Figure 4).

**Alpha1 band**. The prefrontal nodes are driven by posterior nodes in the RG. This is more evident within the right hemisphere (Figure 4).

**Alpha2 band**. The results of the alpha2 connectivity do not have as obvious a pattern as the slower frequency bands. Notably, the left LTC is driven by four other nodes of both hemispheres in the RG, in contrast to the CG, where the left LTC is the driver of three out of the four. It is noteworthy that two connections are stronger within the RG, i.e., the inflow to the left LTC from the left dMPFC and from the right HF+ (Figure 5).

**Beta1 band**. Like the delta band, the left hemisphere drives the right hemisphere in the beta1 band. However, in this band, the intrahemispheric connections are more pronounced within the left hemisphere for the RG (Figure 5).

**Beta2 band**. Interestingly, the connections that differed between the RG and the CG involved the four nodes of the bilateral MPFC. Both dMPFCs are drivers of the vMPFCs, and the left hemisphere seemed to drive the right hemisphere in the RG (Figure 5).

**Beta3 band**. As with other frequency bands, interhemispheric connections involve the left hemisphere driving the right hemisphere in the RG. Additionally, the differences in intrahemispheric connectivity exclusively involved the right hemisphere in the RG (Figure 5).

## Discussion

The results of the present study add to the very little literature available regarding healthy elderly at risk of cognitive decline. The experimental group in the present study consisted of a sample of healthy elderly individuals with an excess of theta activity. We considered an excess of theta activity as an electroencephalographic marker of risk of cognitive decline (8, 75). An increase in slow-wave activity (i.e., delta and theta) is characteristic of MCI and AD (13, 75–79), and has been associated with cholinergic dysfunction (80). Furthermore, Fernández et al. (81, 82) compared patients with cognitive decline to healthy elderly individuals using MEG and found a negative association between slow-wave activity in posterior areas and both their cognitive status and hippocampal volume. Additionally, theta activity has been associated with hypoperfusion (83, 84) and hypometabolism (85) using neuroimaging tools. In AD patients, a relationship between biomarkers of neurodegeneration in CSF, such as a decrease in β-amyloid_1 − 42_ and an increase in p- and t-tau, has been linked to decreased alpha and beta activities (86).

Considering this, we sought to characterize the functioning of the DMN in a group of healthy elderly at risk of cognitive decline by means of its comparison to a group of healthy elderly with normal EEG. To interpret our findings, we assumed that the normal connectivity patterns were those observed in the CG, since they exhibited normal values in their quantitative EEG measures of absolute and relative power and resting-state EEGs provide information about the integrity of an individual's brain functioning (87). Therefore, any difference in connectivity patterns between the CG and the RG could indicate the presence of important and, possibly, pathological conditions.

We observed an increase in connectivity strength in slow frequencies (corresponding to the delta and theta bands) in the RG compared to the CG. The increase in delta and theta connectivity strengths has been previously described when comparing groups of older adults to young adults, and it has been suggested that this might reflect a loss of efficiency among brain regions as a result of progressive disconnection. This was supported by the fact that patients with AD show an increase in delta (88, 89) and theta (88–90) connectivity when compared to cognitively normal controls. Furthermore, we observed a decrease in upper alpha connectivity in the RG, which has also been described as a natural consequence of the aging process when comparing young and older adults. Previous studies have also shown a negative relationship between delta connectivity and cognitive performance (91). Theta connectivity, on the other hand, does not seem to have a clear relationship with cognitive performance. While some studies report a negative relationship between these variables [e.g., (91)], others have shown a positive relationship (92, 93). The decrease in upper alpha connectivity is accentuated in patients with AD (90) and MCI (94) when compared to normal controls. This decrease in alpha connectivity has also been related to worse cognitive performance (91, 95). Moreover, Teipel et al. (96) showed a positive relationship between alpha connectivity and the integrity of white matter tracts within the brain.

Regarding the aforementioned results, Gorges et al. (97) proposed a hypothetical model of functional connectivity changes in relation to behavioral performance during the course of a pathological process. According to this model, the first stage of abnormal brain function involves an initial increase in connectivity strength. This increase in connectivity has been explained in two ways: [1] as a consequence of the loss of inhibitory neural influence on the cortex, product of the aforementioned pathological process (98) or [2] as a compensatory mechanism of neural reorganization that recruits more neurons as the neural reserve of the individual depletes, thereby mitigating the adverse effects of the pathological process (in preclinical or in early stages) in that individual and leads to a behavioral performance within expected normal ranges. This latter explanation might be the case for our RG, where the increase in connectivity strength could reflect a compensatory and adaptive response.

A meta-analysis that considered the results of 126 MRI studies found patterns of increased connectivity in traumatic brain injury and multiple sclerosis, as well as hyperconnectivity patterns in Parkinson's disease. In contrast, studies that compared patients with MCI and AD against cognitively normal subjects showed a decrease in connectivity that worsened as the disease progressed (99). This supports the second stage of abnormal brain function of Gorges's model (70). Global neuronal atrophy and loss of tissue in regions of the medial temporal and parietal cortex have long been reported in AD, which imply a reduction in cerebral volume (100). When the loss of neural resources reaches a critical threshold, the increased connectivity is no longer effective for meeting task demands (97). The availability of neural resources plays a pivotal role in the network's response when there is significant neural atrophy (as in AD) since the flow of information through critical hubs might be interrupted and result in overall network malfunction (101, 102). The critical threshold is determined by a combination of factors associated with the cognitive reserve (103) and the location of the network interruption (i.e., the affected hub). Once the critical threshold is reached, the pattern of connectivity deficits will become evident and will progress along with the disease. The increased connectivity strength for the slow bands in the RG may be interpreted as a loss of communicative efficiency among the nodes of the DMN in the RG, which may impact the flow of information within the network.

When we examined the direction of the flow of information among the nodes of the DMN, i.e., their causal interaction, we observed interesting differences between the RG and the CG. There is surprisingly little information about the direction of connectivity among nodes of the DMN during aging, and most studies have used fMRI, which, as previously mentioned, lacks the temporal resolution of EEG and is an indirect measure of brain activity. Luo et al. (104) explored effective connectivity (EC) differences between two groups of healthy older participants, one of which was consisted of carriers of the APOE ε4 allele, a genetic marker of AD. They found significant increases in EC connectivity from the IPC to the PFC in the ε4 group. They also found that connectivity strength was positively related to memory performance but only in the control group. Regarding the results of our present study, an interesting finding was a shift in the direction of these connections in both hemispheres in the RG. Specifically, while the IPCs were drivers of their ipsilateral dMPFC in the CG, the opposite was true for the RG (left hemisphere: theta and beta1; right hemisphere: delta and beta3). This could be the result of a compensatory role of the dMPFC over the IPCs, which would be in line with several models of cognitive aging which emphasize the importance of the prefrontal cortex as the main source of functional scaffolds during aging (105).

Most of the studies that have explored how AD and MCI affect EC within the DMN have found changes in connectivity patterns that involve the PCC/Rsp (106, 107). Zhong et al. (108) described the PCC as an important convergence node of the DMN and found decreased connectivity in this area in AD patients. When we examined group differences involving the PCC/Rsp, we observed that this area was differentially driven by other nodes of the network in most bands in the CG, except for theta frequencies, where PCC/Rsp drives right medial prefrontal areas, and the alpha2 band, where it drives the left dmPFC. The opposite was true in the RG, as the PCC/Rsp drove other nodes and was driven by prefrontal areas. In the RG, prefrontal nodes were drivers of posterior nodes (left IPL and both PCC/Rsp), in contrast to the CG. The PCC/Rsp area is considered to be one of the most interconnected areas of the brain (109, 110). Moreover, the PCC/Rsp and the IPL cortices are major hubs of the DMN, as indicated by patterns of bidirectional interactions (111). Additionally, when considering unidirectional connections, Deshpande et al. (111) described that the prefrontal cortex and the hippocampus receive major inputs from other areas of the DMN, which may reflect processes of cognitive integration. This is in line with what we observed in theta connectivity in the CG, whereas the PFC served as a driver in the RG and, as mentioned above, may reflect a compensatory role of the PFC over the rest of the nodes of the DMN.

A potential limitation of the present study is the use of a limited number of channels, i.e., 19 channels, for the solution of the inverse problem using BC-VARETA. This issue was addressed in the methods section. Furthermore, it is important to emphasize that the average clinician will only have access to a 19-channel device. Several studies have looked into the accuracy of source localization with different numbers of channels and it appears that, when the signal-to-noise ratio is adequate, a 19-channel recording is fairly accurate (112–114), which of course has positive implications in clinical settings. Future research could be aimed at exploring cognitive differences between these two groups using more demanding tests, perhaps with an emphasis on frontal lobe functioning.

The findings of the present study contribute to the existing knowledge by proposing that a similar ongoing pathological process may be underway in healthy elderly individuals with excess theta activity in their EEGs. Moreover, BC-VARETA appears to be a feasible tool and can be used in clinical settings since it involves significantly lower costs than other neuroimaging tools and can be employed using standard 19-leads EEG to assess brain connectivity.

## Data availability statement

The raw data supporting the conclusions of this article will be made available by the authors, without undue reservation.

## Ethics statement

The studies involving human participants were reviewed and approved by Bioethics Committee of the Institute of Neurobiology of the National Autonomous University of Mexico (INEU/SA/CB/109). The patients/participants provided their written informed consent to participate in this study.

## Author contributions

Conceptualization: MG-L, EG-M, and TF. Methodology and investigation: MG-L and TF. Software and formal analysis: EG-M, AA-G, and DP-L. Resources, supervision, project administration, and funding acquisition: TF. Data curation and writing—original draft preparation: MG-L. Writing—review and editing: MG-L, EG-M, DP-L, AA-G, and TF. Visualization: MG-L, EG-M, DP-L, and TF. All authors have read and agreed to the published version of the manuscript.

## Funding

This work was supported by grants IN225414, IN200817, and IT201123 from the Universidad Nacional Autónoma de México (DGAPA-PAPIIT). During the realization of this work, Mauricio González-López (scholarship recipient: 684408) was a beneficiary of the Consejo Nacional de Ciencia y Tecnología (CONACYT) scholarship 608617.

## Conflict of interest

The authors declare that the research was conducted in the absence of any commercial or financial relationships that could be construed as a potential conflict of interest.

## Publisher's note

All claims expressed in this article are solely those of the authors and do not necessarily represent those of their affiliated organizations, or those of the publisher, the editors and the reviewers. Any product that may be evaluated in this article, or claim that may be made by its manufacturer, is not guaranteed or endorsed by the publisher.
